# Complexity of genome evolution by segmental rearrangement in *Brassica rapa *revealed by sequence-level analysis

**DOI:** 10.1186/1471-2164-10-539

**Published:** 2009-11-18

**Authors:** Martin Trick, Soo-Jin Kwon, Su Ryun Choi, Fiona Fraser, Eleni Soumpourou, Nizar Drou, Zhi Wang, Seo Yeon Lee, Tae-Jin Yang, Jeong-Hwan Mun, Andrew H Paterson, Christopher D Town, J Chris Pires, Yong Pyo Lim, Beom-Seok Park, Ian Bancroft

**Affiliations:** 1 John Innes Centre, Norwich Research Park, Colney, Norwich NR4 7UH, UK; 2 National Academy of Agricultural Science, RDA, Suwon, 441-857, Korea; 3 Plant Genomics Institute, Chungnam National University, Daejeon 305-764, Korea; 4 Department of Plant Science, Plant Genomics and Breeding Institute and Research Institute for Agriculture and Life Sciences, College of Agriculture and Life Sciences, Seoul National University, Seoul, 151-921, Korea; 5 University of Georgia, Athens, GA 30602, USA; 6 The J Craig Venter Institute, 9704 Medical Center Drive, Rockville, MD 20850, USA; 7 University of Missouri, Columbia, MO 65211-7310, USA

## Abstract

**Background:**

The *Brassica *species, related to *Arabidopsis thaliana*, include an important group of crops and represent an excellent system for studying the evolutionary consequences of polyploidy. Previous studies have led to a proposed structure for an ancestral karyotype and models for the evolution of the *B. rapa *genome by triplication and segmental rearrangement, but these have not been validated at the sequence level.

**Results:**

We developed computational tools to analyse the public collection of *B. rapa *BAC end sequence, in order to identify candidates for representing collinearity discontinuities between the genomes of *B. rapa *and *A. thaliana*. For each putative discontinuity, one of the BACs was sequenced and analysed for collinearity with the genome of *A. thaliana*. Additional BAC clones were identified and sequenced as part of ongoing efforts to sequence four chromosomes of *B. rapa*. Strikingly few of the 19 inter-chromosomal rearrangements corresponded to the set of collinearity discontinuities anticipated on the basis of previous studies. Our analyses revealed numerous instances of newly detected collinearity blocks. For *B. rapa *linkage group A8, we were able to develop a model for the derivation of the chromosome from the ancestral karyotype. We were also able to identify a rearrangement event in the ancestor of *B. rapa *that was not shared with the ancestor of *A. thaliana*, and is represented in triplicate in the *B. rapa *genome. In addition to inter-chromosomal rearrangements, we identified and analysed 32 BACs containing the end points of segmental inversion events.

**Conclusion:**

Our results show that previous studies of segmental collinearity between the *A. thaliana*, *Brassica *and ancestral karyotype genomes, although very useful, represent over-simplifications of their true relationships. The presence of numerous cryptic collinear genome segments and the frequent occurrence of segmental inversions mean that inference of the positions of genes in *B. rapa *based on the locations of orthologues in *A. thaliana *can be misleading. Our results will be of relevance to a wide range of plants that have polyploid genomes, many of which are being considered according to a paradigm of comprising conserved synteny blocks with respect to sequenced, related genomes.

## Background

The cultivated *Brassica *species, like *Arabidopsis thaliana*, are members of the Brassicaceae family [1]. *Brassica rapa *(*n *= 10) contains the *Brassica *A genome, which is the smallest, at *ca*. 500 Mb [2]. A genome sequencing project is underway http://brassica.bbsrc.ac.uk/. A number of genome analysis studies have shown that the *Brassica *genomes contain extensive triplication, consistent with their having evolved from a hexaploid ancestor [3-5]. Two sequence-level studies, one in *B. oleracea *[6] and one in *B. rapa *[7] have provided further support for the hypothesis of hexaploid ancestry for the *Brassica *species. Recent cytogenetic studies have shown that a distinctive feature of the *Brassiceae *tribe, of which the *Brassica *species are members but *A. thaliana *is not, is that they contain extensively triplicated genomes [8].

An elegant study using sequenced RFLP markers demonstrated that 21 segments of the genome of *A. thaliana*, representing almost its entirety, could be replicated and rearranged to generate a structure approximating that of the *B. napus *genome [9]. In a similarly ground-breaking study, an ancestral karyotype (AK) of *n *= 8 was proposed for the Brassicaceae, which has been related to the *A. thaliana *genome sequence and the structure of the *B. rapa *genome derived by linkage mapping [10]. Thus the genome sequence of *A. thaliana *is being used, either directly via the *B. napus *comparative analysis or indirectly via the AK inferred genome, to inform studies in the *Brassica *species. A complication in such comparative studies is that there are typically multiple orthologues in *Brassica *species for each gene represented in *A. thaliana*, although interspersed gene loss has reduced the number that might be expected in paleohexaploids such as the *Brassica *species [6].

*Brassica *species have been used to study the early responses of genomes to the induction of polyploidy, via resynthesis of *B. napus *by hybridization of *B. rapa *with *B. oleracea*. Such lines display genome instability, which can persist for many generations [11]. Although this is hypothesised to involve homoeologous non-reciprocal translocations, such evolutionary events have not been studied at the sequence level. Indeed, sequence-level studies in *Brassica *to date have focussed on regions that show collinearity between the *Brassica *genome studied and that of *A. thaliana*. Similarly, in comparative studies in grass genomes, which are considered very much in terms of rearranged collinear blocks [12], little attention has been paid to the regions of collinearity breakdown.

We aimed to test the veracity of our present understanding of the evolution of the *Brassica *and Arabidopsis genomes from the AK genome by identifying and sequencing BAC clones containing genomic DNA of *B. rapa *that represent a sample of collinearity discontinuities (CDs) relative to the *A. thaliana *genome. This involved the development of bioinformatics tools and accessing data arising from ongoing activities to sequence the first four of the ten chromosomes of *B. rapa*.

## Results

### Identification of BAC clones putatively containing collinearity discontinuities

We developed a method by which candidate *B. rapa *BAC clones spanning CDs with the Arabidopsis genome could be identified and selected for sequencing. Our starting point was the set of BAC end sequence (BES) data available for the combined libraries from the 'Chiifu' cultivar, which is the subject of the multinational genome sequencing project. Using a strategy opposite to that employed for selection of seed BACs for that programme, we analysed the mate-pairs within the BES data primarily for inferred disruptions in short- to medium-range synteny (up to five-fold of an average BAC insert, *i.e*. 500 kb). We first conducted a BLASTN similarity search against the Arabidopsis genome sequence with all 200,031 individual BES available from 106,144 *B. rapa *BAC clones, these sequences comprising 93,887 mate-pairs and 12,257 singletons. For each BES we recorded the pseudochromosome coordinates of the most significant alignment above a threshold E-value of 10^-30^. Of the clones with both mate-pair BES available, 26,574 (28%) gave mappings with each E-value above this threshold and were therefore amenable to further analysis.

We loaded these pseudochromosome mappings into our own copy of the ATIDB Arabidopsis genome database [13] to enable a programmatic analysis. A Perl script was developed to interrogate the database and to identify associations between non-contiguous regions of the Arabidopsis genome that are linked by a number of disjoined mate-pair mappings and thus produce a list of cognate *B. rapa *BAC clones that might contain discontinuities. The algorithm we used is described in more detail in Methods. Our initial approach took into account several factors; filtering out instances of clone duplications and discounting mate-pair mappings whose DNA strand dispositions differed from the majority. We experimented with a threshold number, over the range of 2-5, of independent mate-pair mappings linking any given pair of bins required to signal an association.

BAC clones potentially representing CDs with the *A. thaliana *genome were thus selected, one from each association identified by three or more BAC clones. These BAC clones were sequenced and annotated, *inter alia*, for similarity to *A. thaliana *gene models and to *B. rapa *BES using BLASTN. Of the 68 sequenced BACs, 38 were found not to contain CDs. In the majority of these (25), the BACs show alignment of multiple gene models from two regions of the *A. thaliana *genome. These pairs of regions of the *A. thaliana *genome are related to each other, representing paralogous segments. The sequences at one end of each *B. rapa *BAC shows the highest similarity to the corresponding gene model from one of the *A. thaliana *genome segments, whereas the sequences at the other end of the *B. rapa *BAC shows the highest similarity to the corresponding gene model from the other *A. thaliana *genome segment. We termed this paralogue conflation. In the remaining cases, there appears to be at one end of the clone a small stretch of inverted sequence or a single gene (or gene fragment) with similarity elsewhere in the *A. thaliana *genome. The remaining 30 *B. rapa *BAC clones contain similarity to two or more collinear runs of multiple *A. thaliana *gene models.

**Figure 1:**
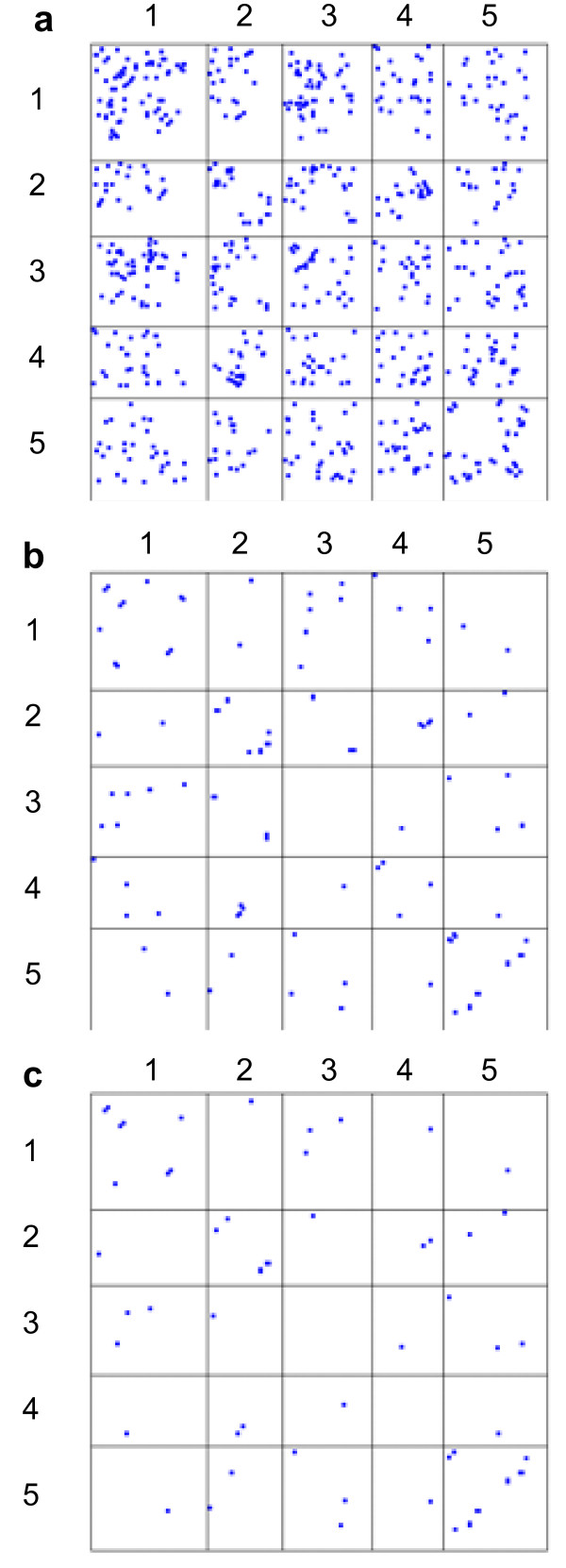
**Representation of candidate collinearity discontinuities within the *A. thaliana *genome with respect to *B. rapa***. The plot is divided into cells with dimensions proportional to the physical lengths of the five Arabidopsis pseudochromosomes (numbered on axes). A blue square within a cell signifies an association between 500 kb bins requiring (a.) 2; (b.) 3 or (c.) 4 distinct instances of disjoined *B. rapa *BES mate-pair mappings linking them.

These findings enabled us to substantially improve the algorithm and to reduce the false positive rate, using the sequenced BACs as a training set. Graphical representations of the results are shown in Figure 1 and a summary is given in Table 1. A majority proportion of the CDs we were seeking would be ancestral to the divergence of the *B. rapa *A genome and *B. oleracea *C genome lineages and so we experimented with adding supplementary data obtained from a *B. oleracea *BAC library. Using identical methods we analysed 85,317 BES derived from 43,691 *B. oleracea *clones, these sequences comprising 41,626 mate-pairs and 2,065 singletons. Of the clones with both mate-pair BES available, 6,623 clones (15.9%) yielded significant mappings at the same E-value threshold as before. This proportion was almost half that obtained from the *B. rapa *dataset, probably due to the higher repeat content in *B. oleracea *reducing the probability of each BES in a mate-pair containing conserved (genic) sequence. When these *B. oleracea *mappings were added and the database re-analysed, the number of associations revealed was increased significantly at each threshold value (Table 1).

**Table 1:** Results of database interrogation for collinearity discontinuities

	*B. rapa *BES		*B. rapa *+ *B. oleracea *BES	
**Threshold**^a^	**Associations**^b^	**Clones**^c^	**Associations**	**Clones**

2	380	893	719	1614

3	54	241	74	324

4	30	169	39	219

5	18	121	23	155

^a^Number of independent BES mate-pair mappings required for an association.

^b^Number of non-contiguous pairs of 500 kb bins linked by mate-pair mappings.

^c^Number of clones linking bins.

### Sequence validation of putative collinearity discontinuities

**Figure 2:**
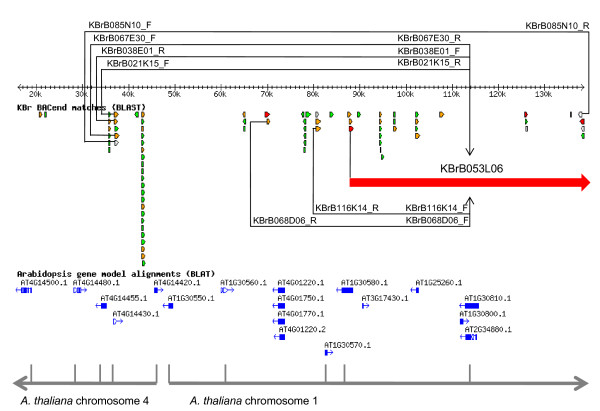
**Validation of collinearity discontinuities**. Annotation of the sequence of BAC KBrB026E16 showing aligned BAC end sequences and homologous *A. thaliana *gene models. Sequence similarities with *A. thaliana *gene models are shown with blue arrows. Sequence similarity with end sequences derived from other *B. rapa *BAC clones are colour-coded: red for the end sequences of fully sequenced BACs, white for the end sequences of BACs for which both can be found within the annotated BAC, orange for end sequences which may bridge to another fully sequenced BAC, and green for other cases. The two inferred collinearity blocks are indicated by grey arrows. The collinearity discontinuity (CD) is confirmed by congruous end sequence alignments of KBrB085N10, KBrB067E30, KBrB038E01 and KBrB021K15. Both end sequences of KBrB085N10 align to KBrB026E16, with the expected orientation of the reads (one in each collinearity block). For each of the remaining three, one end aligns to the *A. thaliana *chromosome 4-related region and the other to regions of KBrB053L06 (shown in red) that extend beyond KBrB026E16, as do one end of each of KBrB068D06 and KBrB116K14 (from which the other ends align to the chromosome 1-related region. Thus multiple BACs (for which only end sequence is available) confirm the CD identified in the sequenced clone, in addition to the fully-contained BAC (KBrB085N10).

A genome sequencing project is underway for *B. rapa*, with sequences derived using a BAC-by-BAC strategy and with annotation being made publicly available http://brassica.bbsrc.ac.uk/. During the course of this effort, we sequenced a number of BAC clones (and sets of overlapping clones) containing CDs relative to the *A. thaliana *genome. Several of these had not had both mate-pair BES available previously and hence would have been undetectable by our computational analysis. These clones, along with those identified from the genome-wide analysis, were then assessed rigorously in order to exclude any that might not be representative of the genome (*e.g*. chimaeric ligation or the result of rearrangement in *E. coli*). This was done by using the sequence annotation to assess whether there are additional *B. rapa *BAC clones overlapping and confirming the discontinuity. This process involved the identification, based on congruous BAC end sequence alignments, of additional BAC clones that confirm the CD, and is illustrated in Figure 2. This resulted in the confirmation of a further 20 discontinuities represented in sequenced BAC clones, making a total of 50, as listed in Table 2. Relative to the *A. thaliana *chromosome structure, 19 of these represent inter-chromosomal rearrangements, with the remaining 31 representing the end points of intra-chromosomal rearrangements (segmental inversions).

**Table 2:** Characterization of *B. rapa *BAC clones containing discontinuities in collinearity with the *A. thaliana *genome

BAC	Sequence Accession	Collinearity with A. thaliana*	Linkage group	**No. confirming BACs**^d^
KBrH106N09	CU915572	4g03430-4g03630| 4g05460-4g05430| 3g26280-3g26570	A9^b^	3

KBrB089J13	CU695271	4g38560-4g38350| 4g14145-4g14350| 1g30480-1g30400	A8^a^	7

KBrH110M01	AC237306	2g22840-2g20920| 2g41990-2g42005| 5g49760-5g49900	A3^b^	2

KBrH013F08	CU695290	3g52930-3g52770| 1g02080-1g01980| 5g42100-5g42020		4

KBrB028F11	AC237302	2g20440-2g20900| 3g24620-3g25290| 1g62200-1g63390	A9^b^	2

KBrB026E16	CU914552	4g14550-4g14420| 1g30550-1g30810	A8^b^	4

KBrH109L07	CU695323	3g49370-3g49660| 5g61770-5g61780	A6^a^	5

KBrH010M06	AC237305	3g49870-3g49670| 5g61760-5g61580	A3^b^	3

KBrH004M24	AC232396	2g01210-2g01060| 5g47810-5g47540	A6^b^	4

KBrB026A12	AC237301	5g47680-5g47760| 2g01110-2g01410	A9^b^	5

KBrH131P10	FP245487	3g24350-3g24495| 1g62120-1g61890	A1^b^	4

KBrH001J23	CU695282	3g49700-3g49730| 1g21900-1g21650	A6^a^	5

KBrH034P16	CU740088	3g52490-3g52500| 4g15230-4g15410	A3^a^	8

KBrH071G03	FP236364	5g42920-5g42470| 4g39770-4g39480		7

KBrH024O16	CU695293	5g49960-5g49810| 1g64970-1g65200		5

KBrB055E21	AC232500	3g01590-3g02000| 4g00050-4g00370	A3^b^	14

KBrH004I22	AC237303	2g04740-2g04050| 3g25520-3g25550	A3^b^	5

KBrH006I08	AC237304	3g63240-3g63460| 2g26540-2g26260	A9^b^	3

KBrH026A01	CU928183	2g25290-2g26170| 4g00040-4g00080| 2g24690-2g24450		2

KBrH066L21	CU695309	5g28060-5g28150| 5g30510-5g28490| 5g49570-5g49360	A9^a^	12

KBrB129C20	CU695281	5g22750-5g22980| 5g60800-5g60460	A3^a^	18

KBrH055O17	FP565592	5g23100-5g23080| 5g60830-5g61380		10

KBrB011D06	AC232452	2g05540-2g05760| 2g07690-2g05840| 2g11890-2g12480	A3^c^	5

KBrB022J01	CU695257	4g35450-4g35335| 4g23620-4g24120| 4g36130-4g36140	A8^b^	3

KBrH108B16	CU984565	5g59700-5g59650| 5g60110-5g59320| 5g59030-5g59130	A3^a^	8

KBrH064I20	CU695308	4g36760-4g36870| 4g37240-4g36880| 4g37260-4g37410	A8^a^	7

KBrH001C16	CU928050	4g37410-4g37250| 4g36880-4g36910	A1^a^	9

KBrH027B04	CU915252	4g37170-4g37240| 4g36870-4g36660	A1^b^	5

KBrH073M17	CU914555	3g08930-3g08600| 3g10330-3g10650	A3^a^	10

KBrH073M07	CU695313	3g09760-3g10180| 3g08530-3g07980	A3^a^	11

KBrH088K13	CU984562	4g00170-4g00770| 4g01370-4g01270	A3^a^	4

KBrH108F05	CU695322	4g01040-4g00800| 4g01400-4g01450	A3^a^	2

KBrB034C23	AC229604	5g26850-5g27020| 5g27930-5g27520	A9^a^	22

KBrH106B05	CU915253	5g28080-5g27950| 5g27110-5g27380	A6^a^	3

KBrH089D10	FP085567	1g08450-1g07860| 1g07705-1g07720	A8^b^	1

KBrH056E23	CU695304	1g13380-1g13400| 1g11860-1g11490	A6^a^	3

KBrB026M01	CU695258	1g23820-1g24180| 1g27210-1g26970	A8^b^	6

KBrB122B06	FP236659	1g49630-1g49590| 1g49880-1g50220		2

KBrB100G10	CU695273	1g50380-1g50980| 1g51420-1g51380		8

KBrB092C03	CU984555	1g65580-1g64750| 1g67220-1g67330	A7^a^	7

KBrH061M05	CU695307	2g32870-2g32830| 2g33120-2g33710	A3^a^	4

KBrH127P20	CU695332	2g46300-2g46140| 2g40430-2g40700	A5^a^	8

KBrH083O14	FP325102	2g47250-2g47950| 2g29950-2g29320		8

KBrB037J21	CU984548	3g24040-3g23940| 3g24300-3g24310	A1^a^	2

KBrB008F10	CU695255	4g17460-4g17480| 4g28380-4g28070	A1^b^	2

KBrB045C11	FP236657	4g20200-4g20890| 4g21710-4g21680		1

KBrB090F01	CU915574	4g39980-4g40100| 4g38060-4g37870	A1^a^	9

KBrB107K13	CU695275	5g02800-5g02030| 5g01040-5g01075	A2^a^	11

KBrB073M23	CU915573	5g02880-5g03370| 5g02580-5g02310		5

KBrH100O23	FP017268	4g03240-4g03610| 4g05460-4g04940		5

* Collinear runs of gene model homologies indicated "-"; discontinuities in collinearity indicated "| "

^a ^Determined by linkage mapping

^b ^Identified during chromosome sequencing activities

^c ^Determined by linkage mapping of an overlapping BAC

^d ^In addition to the sequenced BAC

### Inter-chromosomal rearrangements

Fifteen of the 19 inter-chromosomal CDs were genetically mapped in the *B. rapa *genome, either by direct linkage mapping or by sequence overlap with a BAC mapped by linkage mapping described elsewhere [14], as summarised in Table 2. These could be related to the position in the *Brassica *A genome of CDs previously inferred by linkage mapping-, defined relative to *A. thaliana *chromosomes [9] and subsequently to the AK [10]. We will use the nomenclature At(chromosome number, letter) to refer to the previously described *A. thaliana *chromosome blocks (*e.g*. At1A refers to *A. thaliana *chromosome 1, block A) as described in [9] and AK(letter) to refer to the ancestral karyotype blocks (*e.g*. AKA refers to ancestral karyotype block A), as described in [10].

**Figure 3:**
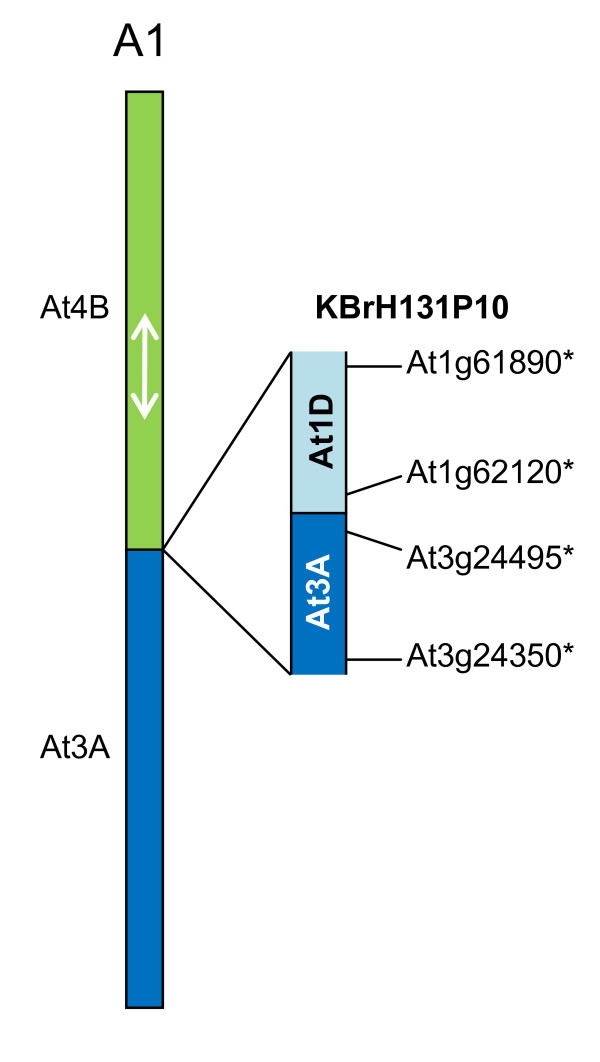
**Collinearity discontinuities mapped to *B. rapa *linkage group A1**. The sequenced BAC clones are illustrated in relation to the CDs with which their mapping is consistent, as represented in [9]. Orthologues delineating the boundaries of recombination events and the end-most aligned gene models within the BACs are designated by the name of the *A. thaliana *gene model suffixed "*".

One sequenced CD (represented by BAC KBrH131P10) mapped to linkage group A1 and is consistent with the position of the inferred CD between blocks At3A and At4B (AKF and AKT), as illustrated in Figure 3. However, the transition revealed by the BAC sequence is between the expected part of At3A (AK block F) but with At1D (AKD). The linkage mapping study [9] had identified markers on A1 with similarity to this region of the *A. thaliana *genome, but there was insufficient evidence to call the block. Only one copy of AKD had been identified previously [10], so our study has identified the position of one of the "missing" two blocks in the *B. rapa *genome that would be expected from its paleohexaploid ancestry.

**Figure 4:**
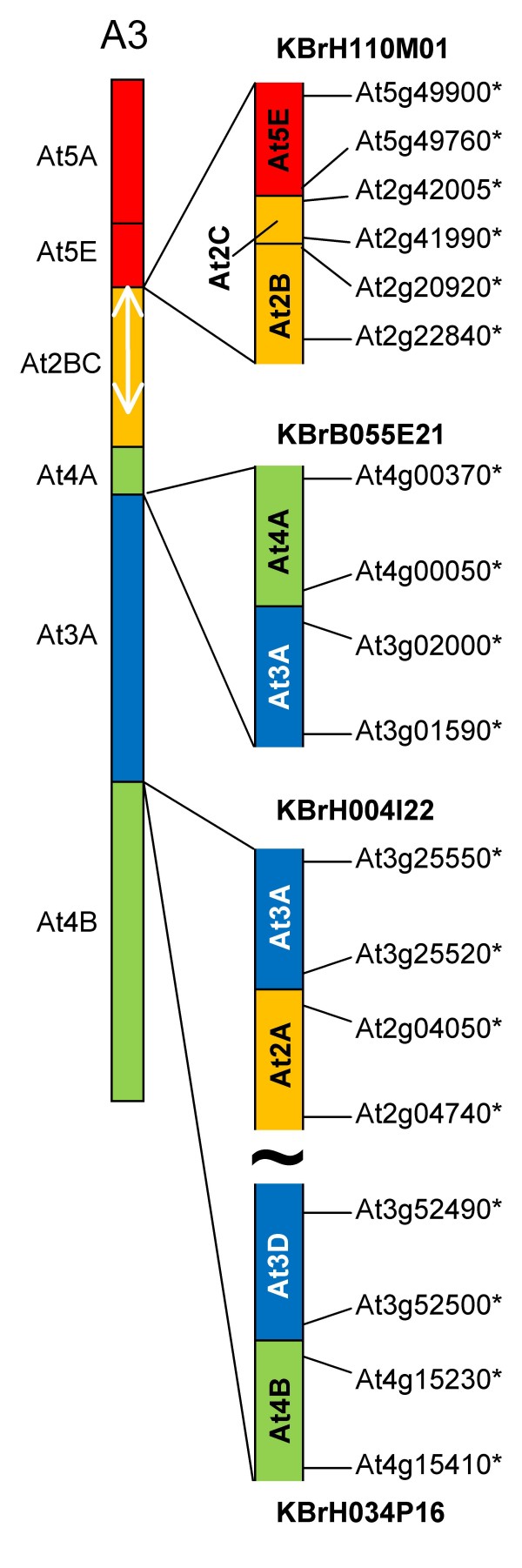
**Collinearity discontinuities mapped to *B. rapa *linkage group A3**. The sequenced BAC clones are illustrated in relation to the CDs with which their mapping is consistent, as represented in [9]. Orthologues delineating the boundaries of recombination events and the end-most aligned gene models within the BACs are designated by the name of the *A. thaliana *gene model suffixed "*".

Five sequenced CDs (represented by BACs KBrH110M01, KBrH010M06, KBrH034P16, KBrB055E21 and KBrH004I22) mapped to linkage group A3, as illustrated in Figure 4. The position of KBrH110M01 is consistent with the position of the inferred CD between At5E and At2B/C (AKW and AK J). However, the transition revealed by the BAC sequence shows an additional small segment in between these, with collinearity to a more distant (internal) part of At2B/C (AKJ). This structure may be the result of rearrangements within this collinearity block.

The sequences within KBrH010M06 represent the end of collinearity block At3C (AKM) and sequences internal to collinearity blocks At5F (AKX), but neither had been identified previously on linkage group A3.

The sequences within KBrH034P16 are internal to collinearity blocks At3D and At4B (AKN and AKT). Although At4B (AKT) had been identified previously on *B. rapa *linkage group A3, At3D (AKN) had not. Therefore the transition previously inferred on this linkage group between collinearity blocks At4B (AKT) and At3A (AKF) [9,10] may be more complex than anticipated. This is supported by the results of analysis of the structure of the *Brassica *A genome as represented in *B. juncea*, in which AKN and AKG-H were identified between AKT and AKF [15].

The sequences within KBrB055E21 represent the ends of collinearity blocks At3A and At4A (AKF and AKO). This is consistent with the transition inferred on the basis of linkage mapping [9], but is not consistent with the inferred interpolation of AKP between AKF and AKO that has been proposed [10].

The sequences within KBrH004I22 correspond to the end of At3A (AKF) and sequences internal to At2A (or at the end of AKK). Although At3A (AKF) had been identified previously on linkage group A3, At2A (AKK) had not. Only two copies of AKK had been identified previously [10], so our study may have identified the position of the "missing" third block in the *B. rapa *genome that would be expected from its paleohexaploid ancestry. The linkage mapping study [9] had identified markers on A3 that have similarity to this region of the *A. thaliana *genome, but there was insufficient evidence to call the block.

**Figure 5:**
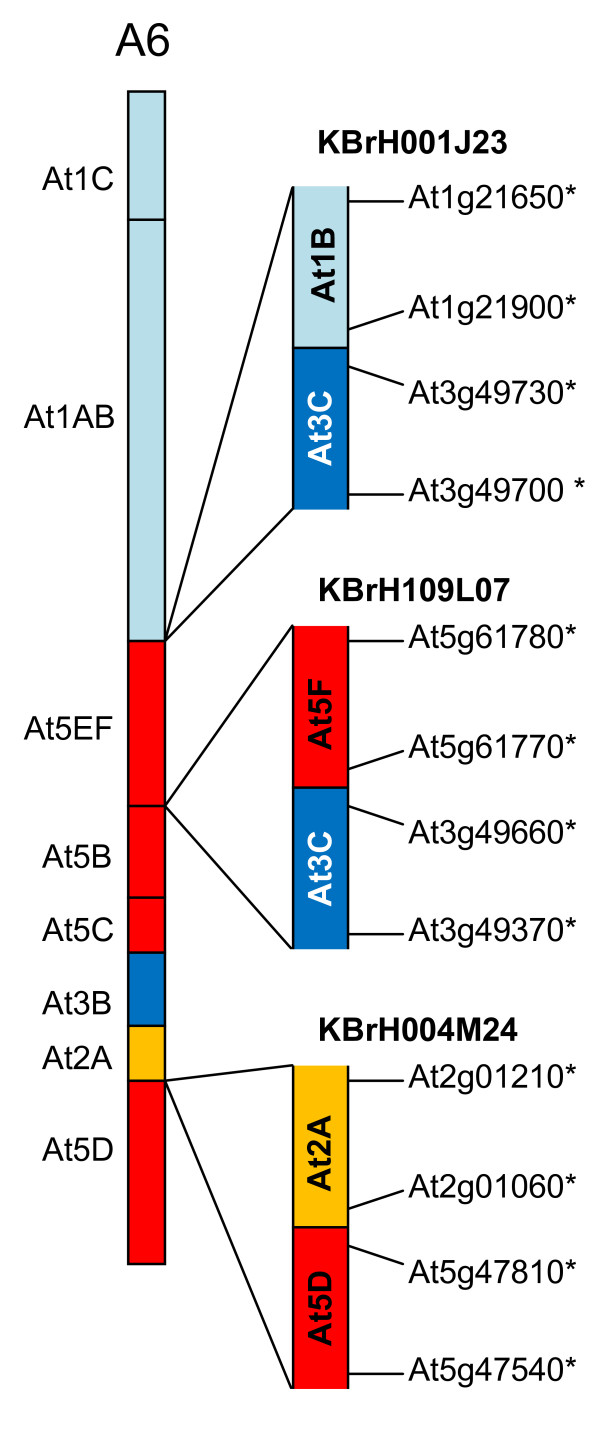
**Collinearity discontinuities mapped to *B. rapa *linkage group A6**. The sequenced BAC clones are illustrated in relation to the CDs with which their mapping is consistent, as represented in [9]. Orthologues delineating the boundaries of recombination events and the end-most aligned gene models within the BACs are designated by the name of the *A. thaliana *gene model suffixed "*".

Three sequenced CDs (represented by BACs KBrH109L07, KBrH004M24 and KBrH001J23) mapped to linkage group A6, as illustrated in Figure 5. The sequences in KBrH109L07 represent the end of collinearity block At3C (AKM) and sequences internal to collinearity blocks At5F (AKX). Block At5F (AKX) had been position on linkage group A6 previously, but no copy of At3C (AKM) had been positioned previously on this linkage group. Thus this BAC represents the position of an additional copy of this segment (along with that identified in BAC KBrH010M06). The linkage mapping study [9] had identified markers on A6 that have similarity to this region of the *A. thaliana *genome, but there was insufficient evidence to call the block.

The sequences within KBrH004M24 correspond to the end of At2A (AKK) and sequences internal to At5D (or at the end of AKV) and confirm one of the CDs on linkage group A6 previously inferred [10].

The sequences within KBrH001J23 correspond to the end of collinearity block At3C (AKM) and sequences internal to collinearity blocks At1B (AKB). Block At1B (AKB) had been position on linkage group A6 previously, but no copy of At3C (AKM) had been positioned previously on this linkage group. Thus this BAC represents the position of the third copy of this segment (along with those identified in BACs KBrH010M06 and KBrH109L07).

**Figure 6:**
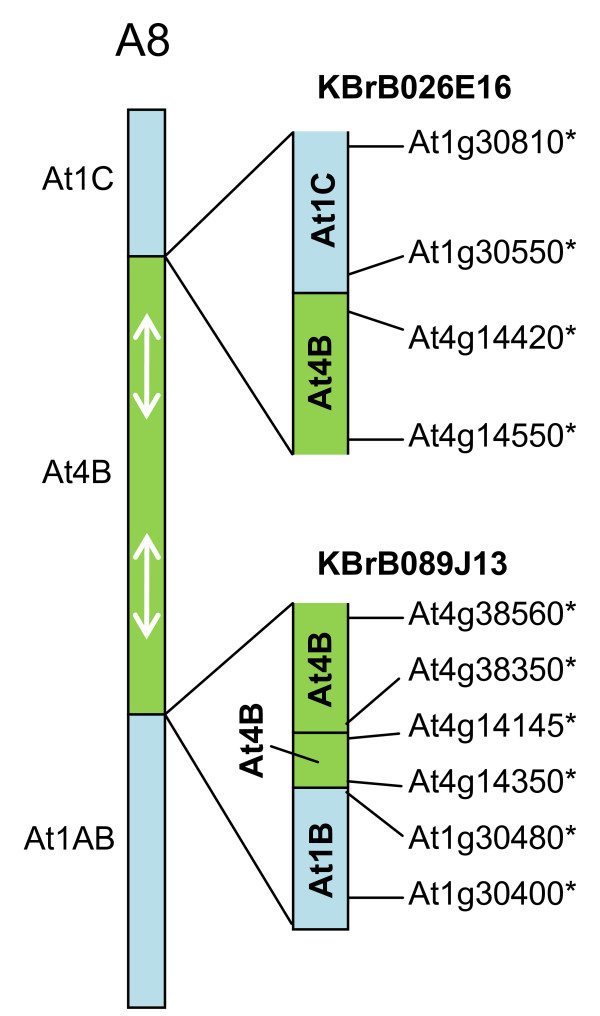
**Collinearity discontinuities mapped to *B. rapa *linkage group A8**. The sequenced BAC clones are illustrated in relation to the CDs with which their mapping is consistent, as represented in [9]. Orthologues delineating the boundaries of recombination events and the end-most aligned gene models within the BACs are designated by the name of the *A. thaliana *gene model suffixed "*".

Two sequenced CDs (represented by BACs KBrB089J13 and KBrB026E16) mapped to linkage group A8, as illustrated in Figure 6. The sequences in both correspond to those close to the ends of collinearity blocks At4B (AKT) and At1B (AKB). Thus they are both candidates for representing the CDs on linkage group A8 previously inferred [9,10]. However, the transition revealed by the sequence of KBrB089J13 shows an additional small segment in between these, with collinearity to a more distant (internal) part of At4B (AKT). This structure may be the result of rearrangements within this collinearity block.

**Figure 7:**
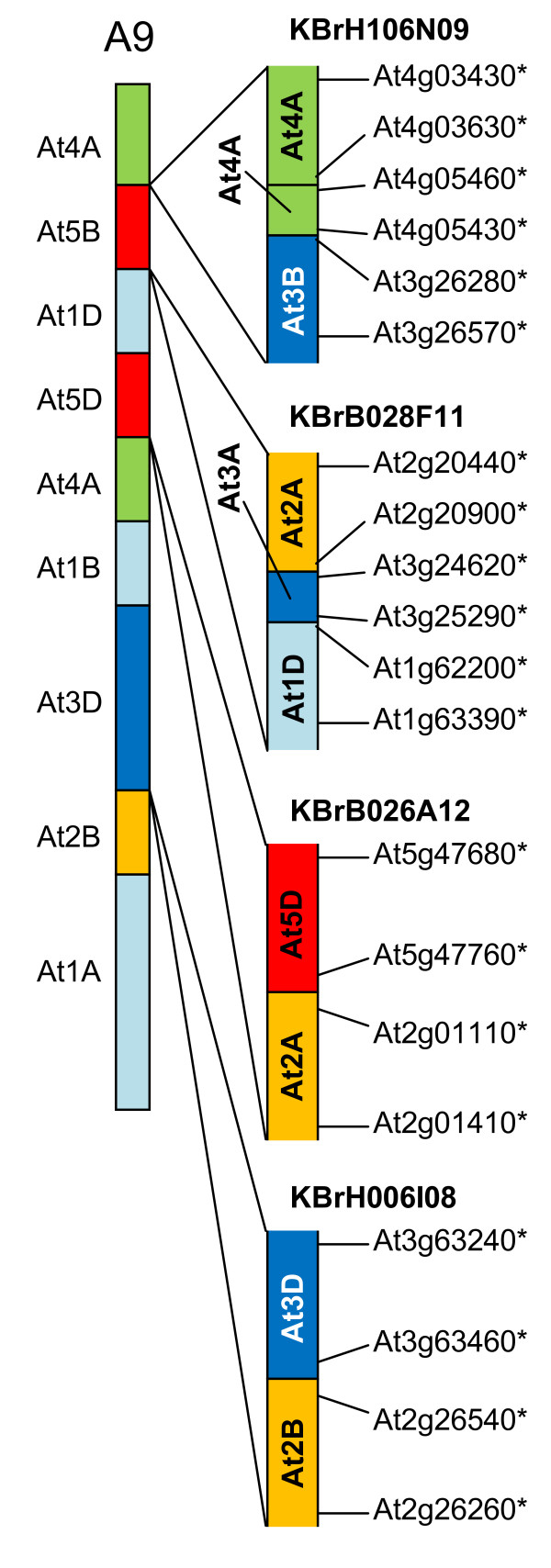
**Collinearity discontinuities mapped to B. rapalinkage group A9**. The sequenced BAC clones are illustrated in relation to the CDs with which their mapping is consistent, as represented in [9]. Orthologues delineating the boundaries of recombination events and the end-most aligned gene models within the BACs are designated by the name of the *A. thaliana *gene model suffixed "*".

Four sequenced CDs (represented by BACs KBrH106N09, KBrB028F11, KBrB026A12 and KBrH006I08) mapped to linkage group A9, as illustrated in Figure 7. The sequences in KBrH106N09 represent sequences within collinearity block At4A (AKO) and within At3B (AKL). Although At4A (AKO) had been identified previously on linkage group A9, At3B (AKL) had not. Only two copies of AKL had been identified previously [10], so our study has identified the position of the "missing" third block in the *B. rapa *genome that would be expected from its paleohexaploid ancestry. There is an additional small collinear segment between AKO and AKL, corresponding to sequences anticipated to have been positioned between AKO and AKP, suggesting that the previously defined boundary of AKO (corresponding to an orthologue of At4g04995) [10] may have been incorrect, with this block extending to an orthologue of A4g05460.

The sequences in KBrB028F11 represent sequences at the end of collinearity blocks At2A (AKH) and within At1D (AKD). Although At1D (AKD) had been identified previously on *B. rapa *linkage group A9, At2A (AKH) had not. Therefore the transitions previously inferred on this linkage group bordering block At1D (AKD) [9,10] may be more complex than anticipated. This is supported by the results of analysis of the structure of the *Brassica *A genome as represented in *B. juncea*, in which AKH was identified as being adjacent to AKD [15]. The linkage mapping study [9] had identified markers on A9 that have similarity to this region of the *A. thaliana *genome, but there was insufficient evidence to call the block. The transition revealed by the BAC sequence shows an additional small segment in between At2A (AKH) and At1D (AKD), with collinearity to the end of At3A (AKF).

The sequences in KBrB026A12 represent sequences at the end collinearity block At2A (AKK) and within At5D (AKV). Although At5D (AKV) had been identified previously on linkage group A9, the part of At2A corresponding to AKK had not. Therefore the transitions previously inferred on this linkage group bordering block At5D (AKV) [9,10] may be more complex than anticipated. Only two copies of AKK had been identified previously [10], so our study has identified the position of the "missing" third block in the *B. rapa *genome that would be expected from its paleohexaploid ancestry. The linkage mapping study [9] had identified markers on A9 that have similarity to this region of the *A. thaliana *genome, but there was insufficient evidence to call the block.

The sequences in KBrH006I08 represent sequences at the end of collinearity blocks At3D (AKN) and within At2B (AKI). They confirm one of the CDs on linkage group A9 previously inferred [9,10], but indicate that At2B (AKI), as represented on linkage group A9, may be truncated.

### Segmental inversions

Twenty four of the 31 CDs representing the end points of intra-chromosomal rearrangements (segmental inversions) were mapped in the *B. rapa *genome, either by direct linkage mapping or by overlap with a BAC mapped by linkage mapping. Their occurrence appears genome-wide, as summarised in Table 2. Few such rearrangements had been inferred previously, and these had not been clearly defined. The positions on linkage group A8 of BAC clones KBrB022J01 and KBrH064I20, and on linkage group A1 of BAC clones KBrH001C16, KBrH027B04, KBrB008F10 and KBrB090F01 are consistent with those expected for the inversions noted in At4B segments on these chromosomes [9].

**Figure 8:**
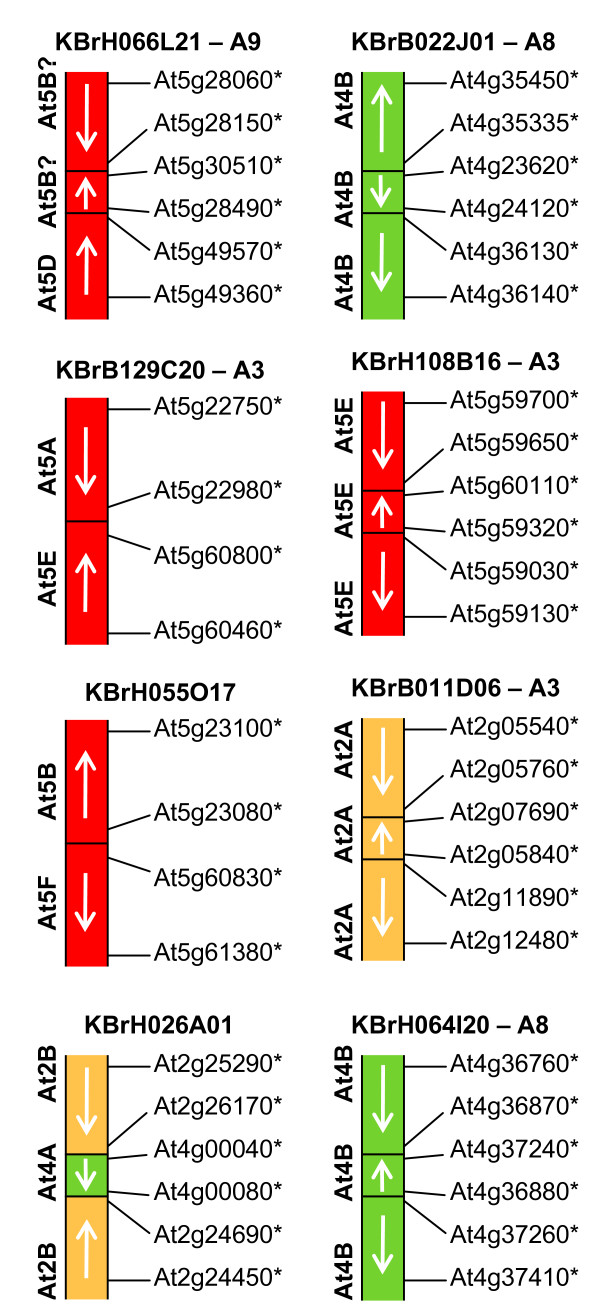
**Collinearity discontinuities involving segmental inversions**. Orthologues delineating the boundaries of recombination events and the end-most aligned gene models within the BACs are designated by the name of the *A. thaliana *gene model suffixed "*". The linkage group to which each BAC maps, where determined, is shown next to the BAC name.

Some of the CDs classified as segmental inversions on the basis of the relationships of genes represented in the BACs to their orthologues in *A. thaliana *may have actually represented inter-chromosomal rearrangements, but segments of those ancestral chromosomes have subsequently come together in the *A. thaliana *genome. This is most notably the case for *A. thaliana *chromosome 5, for which we have three "segmental inversion" CDs spanning relatively large regions of the chromosome. These three are represented by KBrH066L21, KBrB129C20 and KBrB055O17, and are illustrated in Figure 8. The sequences in KBrH066L21, which has been mapped to linkage group A9, represent sequences near the end of collinearity block At5D (AKV) and between At5B and At5C (AKQ and AKS). Both At5D (AKV) and At5B (AKQ) had been identified on this linkage group previously, but not adjacent to each other. This suggests that the transitions previously inferred on this linkage group bordering these collinearity blocks [9,10] may be more complex than anticipated. The identification of sequences corresponding to a region between AKQ and AKS suggests that the previously defined boundary of AKQ (corresponding to an orthologue of At5g28897) [10] may have been incorrect, with this block extending to an orthologue of A5g30510. The distal part of this extended collinearity block AKQ contains a small segmental inversion (relative to the *A. thaliana *genome) that is wholly contained within the BAC. The sequences in KBrB129C20 represents a CD between the ends of collinearity blocks At5E and At5A (AKW and AKR) and is consistent with the position of a previously described CD on linkage group A3 [9,10], to which the clone maps. The sequences within KBrB055O17 represents a CD between At5F and At5B (AKX and AKQ), respectively, but we were unable to determine its position in the genome.

In addition to the small segmental inversion (relative to the *A. thaliana *genome) contained within KBrH066L21, we found further examples of secondary rearrangements at the points of CDs in KBrB022J01 and KBrH108B16, and wholly contained inversions within KBrB011D06 and KBrH064I20, as illustrated in Figure 8. We identified one example of a CD apparently representing intra-chromosomal rearrangements that were separated by sequences from elsewhere in the genome. The sequences in KBrH026A01 represent a small segment from the end of At4A (AKO) at one end of a segmental inversion within At2B (AKI), as illustrated in Figure 8.

### Analysis of collinearity discontinuity sequences

None of the BAC clones containing the CDs was found to contain *B. rapa *satellite repeat sequences characteristic of centromeres [16,17], nor was any found with tandem tracts of TTTAGGG repeats that are associated with telomeres [18], although clone KBrH108B16 did have a cluster of 31 such repeats interspersed over 926 bp, but some 17 kb from the CD.

Minimal sequences defining the confirmed CDs were then selected from these BACs by manual inspection, aided by annotated *ab initio *gene predictions and Brassica EST alignments. These CD regions were then compared with the complete set of annotated *B. rapa *genome sequence currently available (approximately 120 Mb of gene space). The results are given in Table 3. The CD sequences appear to have nucleotide compositions intermediate to those of genic and intergenic regions in terms of microsatellite (simple sequence repeat) density. Although the CDs show densities of apparent gene features typical of the averaged genome sequence, detailed analysis reveals that these features are often gene fragments with homology to Arabidopsis gene models from regions outside those brought together at the discontinuities. Furthermore, they are relatively transcriptionally inactive, with the proportion of models with EST support (obtained from alignments with transcript assemblies derived from all available EST sequences) being about half that of the average for predicted genes. The CD regions also appear to be relatively transcriptionally silent when analysed with the very sensitive but targeted method of alignment with Brassica A genome Solexa reads derived from the *B. napus *leaf transcriptome.

**Table 3:** Summary of features over identified collinearity discontinuities and all annotated *B. rapa *sequence

	All sequence	Genic	Intergenic	All CDs	Inter-chromosomal CDs	Intra-chromosomal CDs
%GC content	35.4	40.9	30.1	34.0	35.2	33.3

SSR density/kb	0.22	0.17	0.27	0.24	0.28	0.21

Gene model density/kb	0.23	n/a	n/a	0.20	0.23	0.18

Models with EST support/kb	0.13	n/a	n/a	0.09	0.11	0.08

Mapped Solexa leaf read density/kb	40.8	64.6	12.6	13.7	15.6	12.5

## Discussion

Computational methods were used to successfully identify BAC clones representing verified CDs between the genomes of *B. rapa *and *A. thaliana*, and relative to an ancestral karyotype. Along with CDs identified during the ongoing chromosome sequencing project, these represent a substantial (but incomplete) sampling of the CDs in the genome of *B. rapa*. Previous studies had defined a segmental structure for the paleohexaploid *Brassica *genome based largely on genetic linkage of markers with similarity to sequences in the *A. thaliana *genome [9,10]. Whereas the seminal study in this area [9] compared the arrangements of the *B. napus *genome with that of *A. thaliana*, and ours compared the arrangement of the *B. rapa *genome with that of *A. thaliana*, we anticipate that the results should be directly comparable as there seems to be little difference in the organization of the A genome in these two *Brassica *species [19]. Remarkably few of our CDs correspond to those expected from this structure: 3 of the 18 representing inter-chromosomal rearrangements and 6 of the 32 representing intra-chromosomal rearrangements. The relatively high "noise" inherent to comparative genomics studies in *Brassica *species, which is a consequence of the widespread occurrence of apparently transduplicated fragments of genes [6], means that multiple instance of collinear alignments are required to correctly identify collinear genome segments. This requirement limits the ability to identify relatively small segments using, for example, comparative linkage mapping based on RFLP markers. Although the paleopolyploid ancestry of *Brassica *species is now widely accepted, the lack of discernable triplication throughout the genome has not been fully explained. The hypothesised segments that have not been identified had been assumed to have been deleted. However, we have found evidence for the existence of numerous additional copies of genome segments, bringing the count of many of these to (or closer to) the predicted three. In one case (At3C/AKM) we identified and mapped onto the *B. rapa *genome three copies where none had been identified previously in that species, with only one copy having been identified in the *Brassica *A genome as represented in *B. juncea *[15].

**Figure 9:**
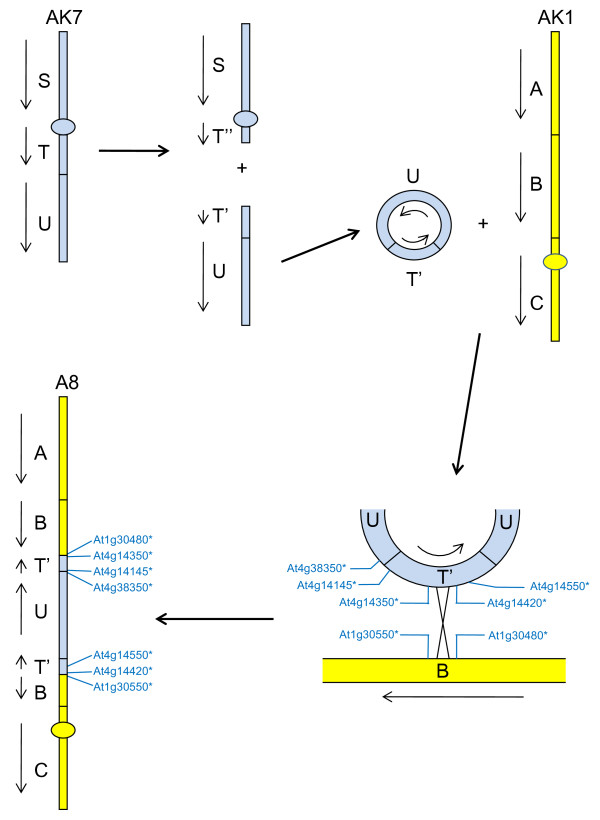
**Hypothetical derivation of *B. rapa *linkage group A8**. Ancestral karyotype segments are labelled (A, B, C, S, T, U) and oriented by arrows. Orthologues delineating the boundaries of recombination events are designated by the name of the *A. thaliana *gene model suffixed "*".

**Figure 10:**
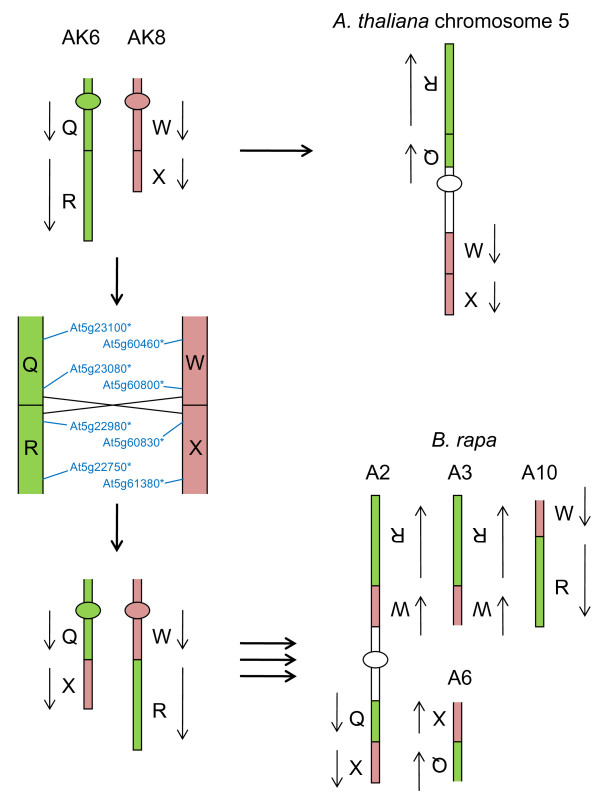
**Hypothetical derivation of *A. thaliana *and *B. rapa *linkage groups from the ancestral karyotype**. Ancestral karyotype segments are labelled (Q, R, W, X) and oriented by arrows. Orthologues delineating the boundaries of recombination events are designated by the name of the *A. thaliana *gene model suffixed "*".

Our analyses of CDs enable us to hypothesise how parts of the genomes of *A. thaliana *and *B. rapa *have evolved from the AK. For *B. rapa *linkage group A8, which contains one copy of AK blocks T-U, our data enable the development of a model for the derivation of the chromosome from the AK, as shown in Figure 9. The detection of sequences from AKT at both ends of an AKU collinearity block indicates that there may have been a circular intermediate derived from linkage group AK7 which was integrated into AK1 to form the progenitor of *B. rapa *A8. Some rearrangements of the AK seem to have taken place in the *Brassica *lineage before genome triplication, but not in the *A. thaliana *lineage. For example, as shown in Figure 10, *A. thaliana *chromosome 5 contains the pairs of AK blocks AKQ-AKR and AKW-AKX (derived from linkage groups AK6 and AK8, respectively). Whereas these AK blocks appear to have been recombined in the ancestor of *B. rapa*. This recombination is represented in *B. rapa *three times [10], on linkage groups A2, A3 and A10 for AKW-AKR, and at least twice for the reciprocal outcome of the recombination, AKQ-AKX (on A2 and A6).

## Conclusion

Our results show that previous studies of segmental collinearity between *A. thaliana*, *Brassica *and AK genomes, although very useful, represent over-simplifications of the true inter-relationships of the genomes. In addition to the occurrence of individual genes in non-collinear regions of the genomes previously noted [6], the presence of numerous cryptic collinear genome segments and the frequent occurrence of segmental inversions mean that inference of the positions of genes based on the locations of orthologues in *A. thaliana *can be misleading. Indeed, excessive reliance on collinearity with the genome of *A. thaliana *may prove problematic for the ongoing efforts to sequence the *B. rapa *genome. Polyploidy is common in plants, and there is no reason to conclude that the greater complexity of segmental rearrangement and evolution that we have observed is unusual. Therefore, our results will be of relevance to studies in a wide range of polyploid plant genomes, many of which are being considered as having blocks of conserved synteny with respect to the genomes of model species, and studies relating to evolutionary breakpoints and their relation to genome organisation [20].

## Methods

### Computational analysis

The 200,031 publicly available BAC end sequence reads (BES) from the combined KBrH, KBrB and KBrS *Brassica rapa ssp. pekinensis *cv. Chiifu libraries, which were provided by the Korea *Brassica *Genome Resource Bank, were used in a WU-BLASTN search [21] versus the TAIR v6 Arabidopsis pseudomolecule sequences, using 1E-30 as the E-value cutoff. Supplementary BLAST parameters used were application of the Dust simple sequence filter and setting hspsepsmax = 1000, appropriate for use against very large subject sequences. Coordinates and scores for individual HSPs from the significant hits were then parsed into GFF format and loaded as features into a local copy of the ATIDB genome database [13] which is built on the GBrowse platform using a MySQL adaptor [22]. An identical exercise was performed with a set of 85,317 *B. oleracea *BES obtained from line TO 1434 and these data added incrementally.

A Perl CGI script was developed to interrogate the ATIDB database using the Bio::DB::GFF applications programming interface and methods. The five Arabidopsis reference chromosome sequences (pseudomolecules) were divided into bins of a selectable size (250 kb - 1 Mb) and the *B. rapa *(and *B. oleracea*) BES features mapping within each were extracted and loaded into a hashed array structure, keyed by chromosome and bin. Each bin was then systematically compared with every other bin, with the algorithm exploiting mirror symmetry for efficiency. Text string comparisons of feature object names (*e.g*. KBrH088K13_F and KBrH088K13_R) were used to identify mate-pairs amongst the BES mappings linking any given pair of bins. The raw mate-pair associations between bins identified from this initial process are inherently noisy and so the algorithm goes on to filter them on a combination of theoretical and empirical criteria. These can be summarised as follows: (1) any mate-pair mappings between neighbouring bins on the same chromosome were discounted if their physical separation in Arabidopsis pseudomolecule space was less than a set threshold of 500 kb, reflecting our estimate of the conserved microsynteny range; (2) duplicate instances of the mate-pair mappings, indicating either simple duplications of clones within libraries or multiple cloning events of the same DNA fragment during library construction, were eliminated; (3) DNA strand dispositions of mate-pair BES mappings (*e.g*. "Chr3:Bin20:plus" *vs*. "Chr5:Bin10:minus") were analysed to eliminate minority variants as it was reasoned that independent physical correlates of CDs should reflect a consistent pattern (unlike chimaeric clones generated by *in vitro *recombination events), and finally, in a development of the algorithm prompted by analysis of false positives; (4) the raw BES mappings in pseudomolecule space were used to locate the nearest annotated gene models and mate-pair mappings were eliminated if these conflicted at either end with the results of direct BLASTN query of the BES against annotated Arabidopsis genes - countering what we termed paralogue conflation;. We imposed an arbitrary threshold for the number of independent mate-pair mappings to annotated gene regions required to trigger a significant association. We varied this threshold between 2 and 5 in order to experiment with the signal to noise ratio in the dataset.

The final output of the script was directed through two routes, a graphical dot-plot style representation of the mate-pair associations using an interface to the GridMap Java applet [23] and also a spreadsheet format summary of the details underlying each significant association (clone identifiers, HSP coordinates and gene models, strand dispositions). The implementation is available from additional file 1 and additional file 2.

### Sequence annotation and analysis

Our automated annotation pipeline [24] was used to analyse the sequences at the CDs. All annotated *B. rapa *BAC sequences were stored in a GBrowse MySQL database [22]. Minimal regions for each CD were manually selected by identifying the sequence flanked by the Arabidopsis gene models listed in Table 2, supplemented (where informative) either by annotated *Brassica *gene predictions from SNAP [25] post-processed with PASA [26] using raw EST data or by BLAT alignments [27] of *Brassica *transcript assemblies [28]. The CD sequences were analysed for the presence of characterised telomeric or centromeric repeats and then scanned for various annotated features with a Perl script using Bio::DB::GFF methods. This was repeated for extracted subsets of the entire annotated sequence defined as genic or intergenic by the gene predictions. Solexa leaf transcriptome reads obtained from *B. napus *[29] were aligned with MAQ [30] onto the *B. rapa *BAC sequences.

## Competing interests

The authors declare that they have no competing interests.

## Authors' contributions

IB conceived of the study, participated in its design and coordination, and helped to draft the manuscript. MT conceived and implemented the CD identification tool and helped to draft the manuscript. ND helped to implement the BAC annotation pipeline. SJK conceived BAC selection, sequencing and identification for CDs of A09 and A03. FF, ES, TJY, JHM and BSP identified and validated the BACs for sequencing. SRC, ZW, SYL and YPL anchored sequenced BACs to the *B. rapa *linkage map. CDT, AHP and JCP contributed to the conception of aspects of the study and contributed for analysis the end sequences of *B. oleracea *BAC clones prior to public release. All authors read and approved the final manuscript.

## Supplementary Material

Additional file 1 **Simple HTML page used to select parameters and options for launching accompanying Perl CGI script (additional file **2**).** Click here for file

Additional file 2 **Perl CGI script used to identify putative collinearity discontinuities (CDs) from BAC end mapping data.** Output is directed to both graphical (requires GridMap applet [23]) and spreadsheet streams. There are numerous code dependencies, mostly commented in the script. The prerequisite is a Bio::DB::GFF database handle to a MySQL (or other) database storing the end mappings defined with respect to the reference sequence. The first author (MT) can assist with technical questions on implementation. Click here for file
