# Supplementary material for: Complexity of genome evolution by segmental rearrangement in Brassica rapa revealed by sequence-level analysis
Source: BMC Genomics. 2009 Nov 18;10:539. doi: 10.1186/1471-2164-10-539 (PMC2783169; doi:10.1186/1471-2164-10-539)
Supplement: Additional file 1 — Simple HTML page used to select parameters and options for launching accompanying Perl CGI script (additional file 2). [file 1471-2164-10-539-S1.HTML]

Breakpoint analysis


# Collinearity discontinuity analysis

---


|  |
| --- |
| Chromosome bin size: 250 kb 500 kb 1 Mb |
| Microsynteny range: only consider intrachromosomal bins separated by greater than 250 kb 500 kb 1 Mb |
| Sequence identity threshold: only consider mappings with better than 60% 70% 80% % identity |
| Apply heuristic filter Log filtering process |
 Paralogue cleanup during filtering: Off  Weak  Strong || Coincidence threshold: min number of (filtered) instances linking bins 2 3 4 5 |

---


For help, comments and bug reports, contact Martin Trick  

Last modified: Thu Jun 25 13:12:20 BST 2009
